# Supplementary material for: Shaping Phenolic Resin-Coated ZIF-67 to Millimeter-Scale Co/N Carbon Beads for Efficient Peroxymonosulfate Activation
Source: Molecules. 2024 Aug 27;29(17):4059. doi: 10.3390/molecules29174059 (PMC11397324; doi:10.3390/molecules29174059)
Supplement: Supplementary file 1 [file molecules-29-04059-s001.zip › molecules-3104464-supplementary.pdf]

Shaping phenolic resin coated ZIF-67 to millimeter scale Co/N carbon beads for efficient peroxymonosulfate activation

Xin Yan, Yiyuan Yao, Chengming Xiao, Hao Zhang, Jia Xie, Shuai Zhang, Junwen Qi*, Zhigao Zhu, Xiuyun Sun, Jiansheng Li*

Jiangsu Key Laboratory of Chemical Pollution Control and Resources Reuse, School of Environmental and Biological Engineering, Nanjing University of Science & Technology, Nanjing 210094, China

E-mail: qijunwen@njust.edu.cn; lijsh@njust.edu.cn

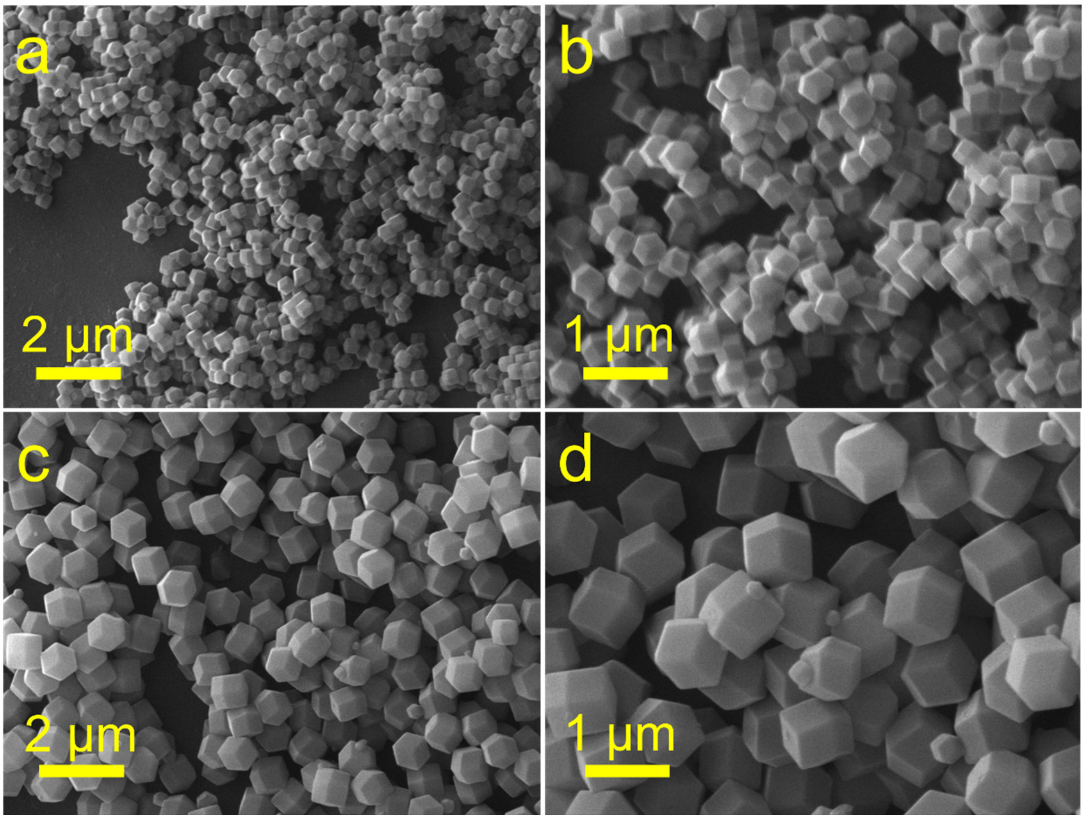

Figure S1. (**a**) and (**b**) SEM images of ZIF67, (**c**) and (**d**) SEM images of ZIF67@AF.

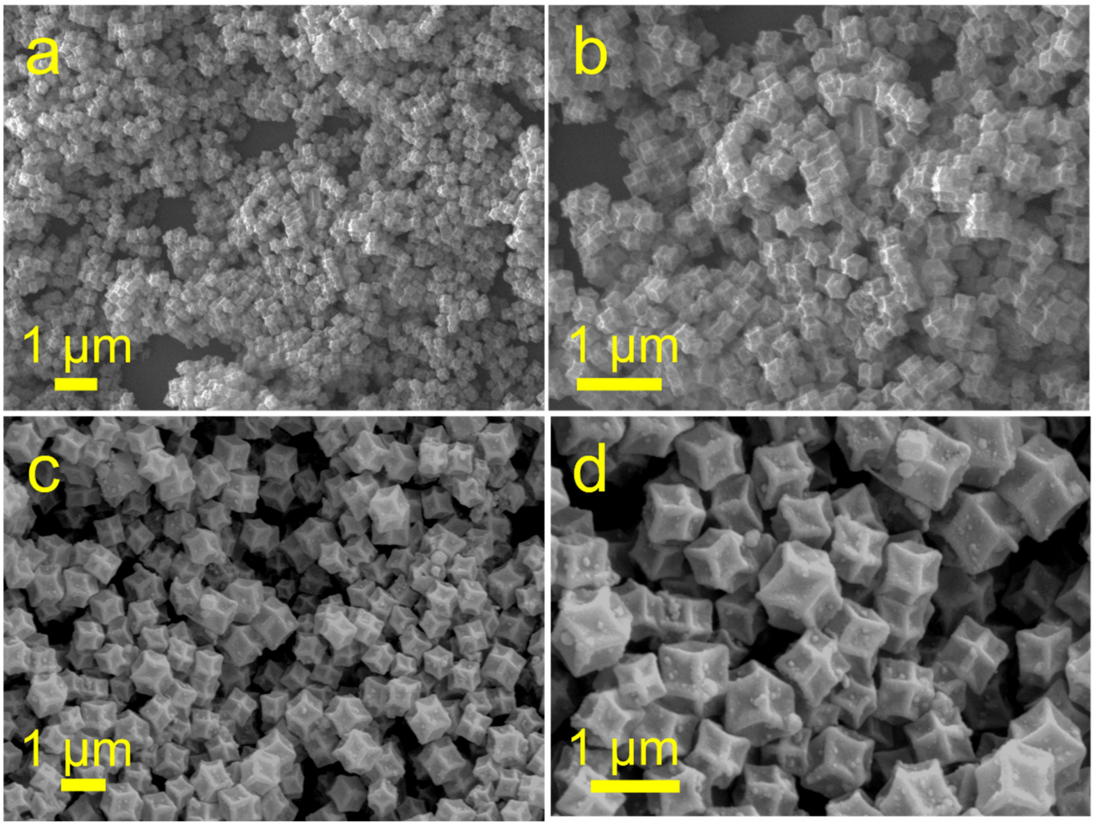

Figure S2. (a) and (b) SEM images of ZIF67 after pyrolysis, (c) and (d) SEM images of ZIF67@AF after pyrolysis.

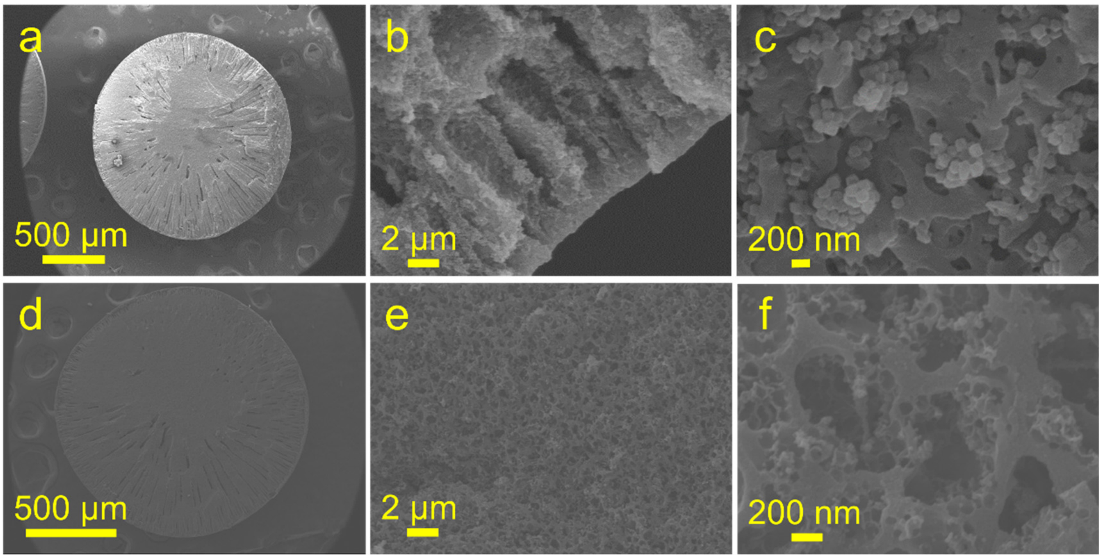

Figure S3. (a) ,(b) and (c) SEM images of ZIF67/PAN beads before pyrolysis, (c) and (d) SEM images of ZCBs after pyrolysis.

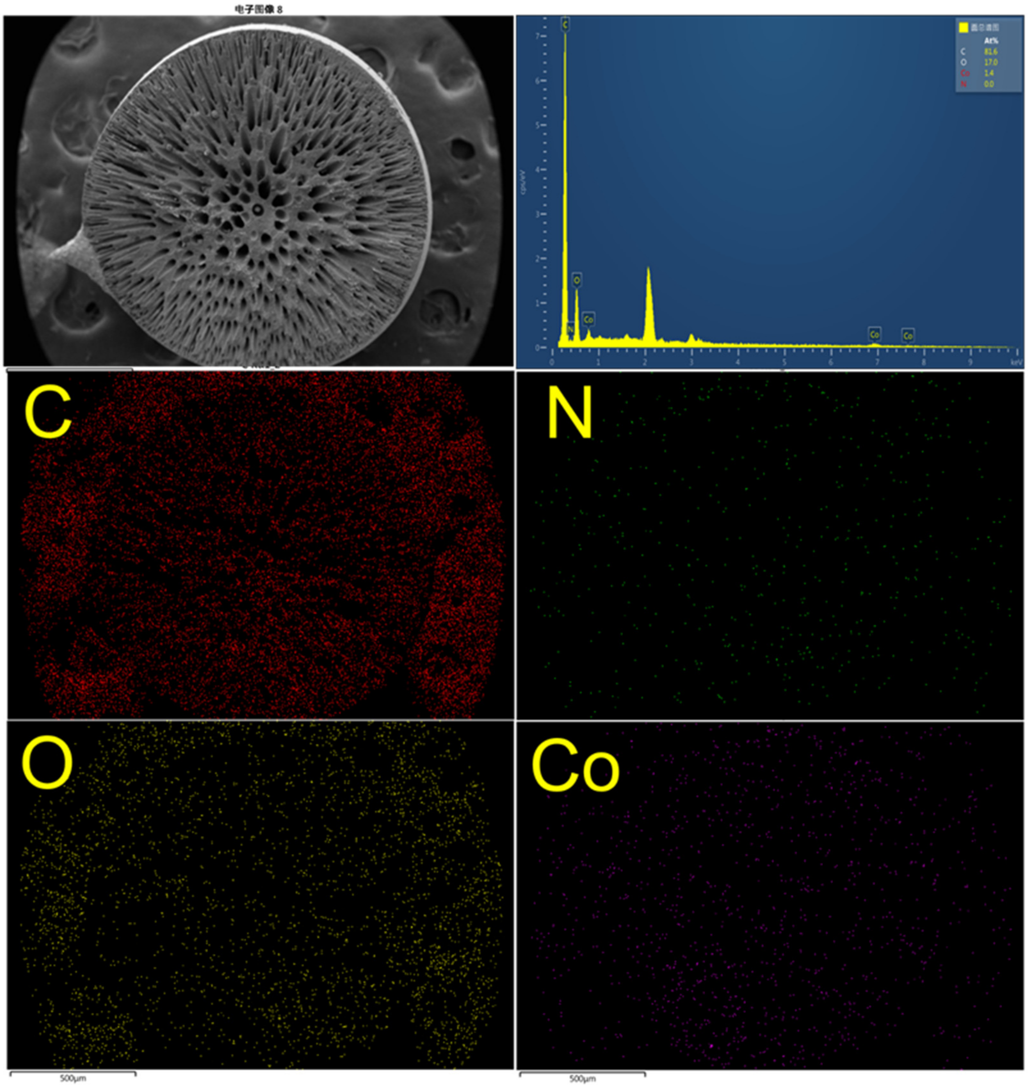

Figure S4. Elemental mapping revealing the elemental distribution of C, N, O and Co in the ZACBs.

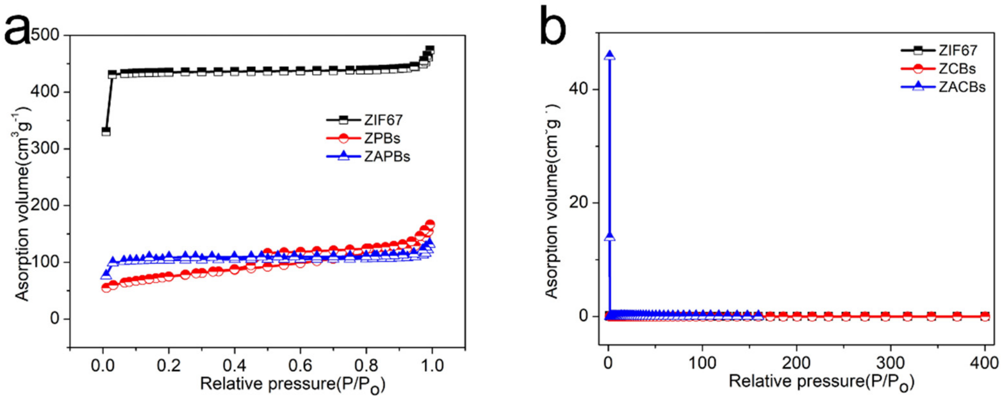

Figure S5. (a) N_2 adsorption/desorption isotherms, and (b) pore size distribution of ZIF67, ZPBs and ZAPBs, respectively.

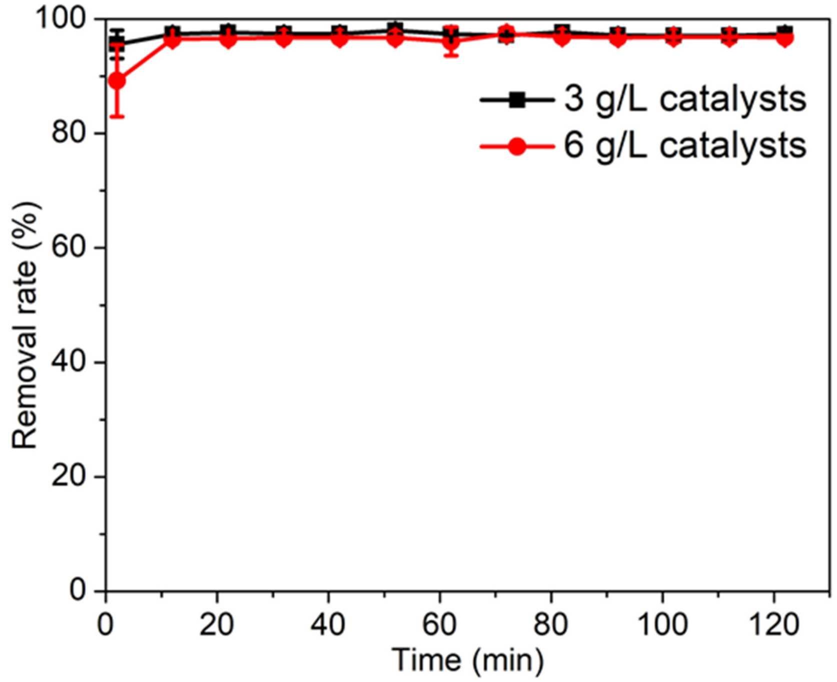

Figure S6. Fixed bed experiment. Experimental conditions: flow rate= 1 mL /min],

[PMS]=0.4 g/L, [TC]=0.3 g/L.

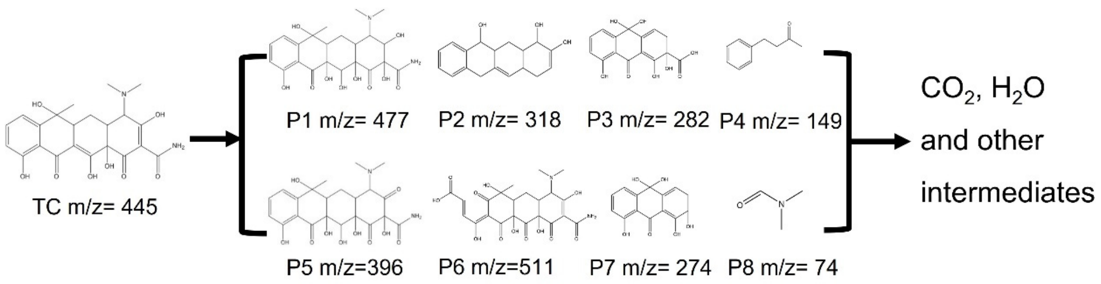

Figure S7. The possible pathway of TC degradation in ZACBs/PMS system.

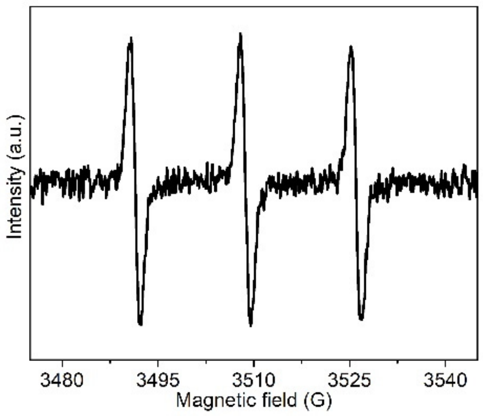

Figure S8. EPR spectra for the detection of $^{1}O_2$.

Table S1. The structural parameters of different samples before carbonization

Materials	ZIF-67	ZPBs	ZAPBs
BET Surface area (m^2/g)	1438.9	435.8	348.1
Pore volume (cm^3/g)	0.73	0.26	0.2

Table S2. The structural parameters of different samples

Samples	S_{BET} (m^2/g)	Pore Volume (cm^3/g)	Crushing strength (N/particle)
ZACBs	304.3	0.31	23.59 ± 2.38
ZCBs	293.4	0.24	9.98 ± 5.83
ZIF-67-C	308.6	0.29	--

Table S3. The comparison of performance of different catalysts

Catalyst	Pollutant(ppm)	PMS(g dm^{-3})	Remove efficiency (%)	Ref.
CoOx-CN	TC(10)	0.3	99.4(21 min)	43
Ru/Co_3O_4@CoBi	TC(20)	0.4	98(14min)	44
CZMO	TC(10)	0.2	97.9(40min)	45
0.4-PAC-8	TC(20)	0.2	86(20min)	46
HPCBs	TC(50)	0.5	85.1(120min)	11
HCNSs-9	BPA(20)	0.15	97(15min)	40
ZACBs	TC(20)	0.2	99(80min)	This work
